# Prevalence and determinants of diabetes-related psychological distress in a tertiary care setting in Tamil Nadu, India: cross-sectional study

**DOI:** 10.1192/bjo.2026.11020

**Published:** 2026-04-27

**Authors:** Gowri Palaniswamy, Lawrence Soosai Nathan, Senthil R. Kumar, Aravindakumar Subramanium, Karthikeyan Aravindakumar Gowri, Dhasaratharaman Thirunavukkarasu, Samuel J. Tromans, Rohit Shankar

**Affiliations:** Department of Diabetology, Kauvery Hospital, Trichy, India; Department of Psychology, Anugraha Institute of Social Sciences, Dindigul, India; Madras Medical College, Chennai, India; Division of Public Health and Epidemiology, University of Leicester, Leicester, UK; Adult Learning Disability Service, Leicestershire Partnership NHS Trust, Leicester, UK; Peninsula School of Medicine, University of Plymouth, Truro, UK

**Keywords:** Psychological distress, mental health, physical illness, diabetes mellitus, liaison

## Abstract

**Background:**

Diabetes distress, whereby people with diabetes experience distressing psychological symptoms associated with living with their condition, is an emerging problem in India. Diabetes distress leads to self-care deficits, suboptimal glycaemic control (which can lead to increasing risks of complications) and impaired quality of life.

**Aims:**

To determine the burden of diabetes distress and its associated factors in an Indian tertiary care centre in Trichy, Tamil Nadu, India, covering a population of 1.25 million.

**Method:**

This prospective observational study involved a structured questionnaire covering demographic and clinical details, which was given to patients. The Diabetes Distress Scale 17 (DDS-17) was used to assess diabetes distress levels. The DDS-17 also measures four subdomains: emotional burden, physician-related distress, regimen-related distress and diabetes-related interpersonal distress. Patients were divided into two groups based on their DDS-17 score: no diabetes distress (DDS-17 score <2) versus diabetes distress (DDS-17 score ≥2) and compared. Correlation analysis, chi-squared tests and *t*-tests were used, with *P* < 0.05 considered statistically significant.

**Results:**

Of 1019 respondents (mean age 56 years; 59.6% male, 40.4% female), diabetes distress was reported in 24.4% (*n* = 249). Factors significantly associated with higher DDS-17 scores were younger age (<45 years) (*P <* 0.0001), long-standing diabetes (>10 years) (*P <* 0.0001), and smoking and alcohol (*P <* 0.05). Significant protective factors for diabetes distress included working, daily exercise, no comorbidities and medical insurance cover (*P <* 0.05). Significant positive correlation between DDS-17 score and all four subdomains was observed (*P <* 0.0001).

**Conclusions:**

Our findings highlight the need for routine psychological screening and holistic management strategies in diabetes care, to improve patient outcomes and quality of life.

Diabetes is becoming an increasingly pressing issue worldwide.^1^ In 2021, there were an estimated 539 million (95% uncertainty interval 500–564 million) people living with diabetes across the globe, with a corresponding global age-standardised total diabetes prevalence of 6.1% (95% uncertainty interval 5.8–6.5).^1^ Around 11% of Indian people are diagnosed with diabetes, with a higher burden in urban compared with rural regions (16 *v*. 8%).^2^ With changes in lifestyle and food habits, this increasing prevalence of diabetes among Indian people shows no sign of slowing down.^3^ Inadequate glycaemic control, adhering to the prescribed diet and lifestyle to manage diabetes, the risk of developing comorbidities and the financial burden of managing the condition affect the individual’s psychological well-being, leading to distress and affecting quality of life.^4^ Although medical management in this group of people is well established, psychological support is inadequate, resulting in poor quality of life and generalised well-being.^5^

## Psychological challenges in people with diabetes

Psychological challenges linked to diabetes include depression, anxiety, eating disorders, schizophrenia, affective disorders, Alzheimer’s disease and phobic reactions.^6–11^ The prevalence of depression has been reported to be 22% in type 1 and 19% in type 2 diabetes, and varied based on factors such as study setting, care type and economic status.^12^ Generalised anxiety disorder had a prevalence rate of almost three times higher than that reported in general population, and about 12–40% of people with type 2 diabetes had an eating disorder.^13,14^ People with diabetes also have a higher incidence of cognitive decline and an increased risk of developing Alzheimer’s disease.^15^

Another challenge among people with diabetes is diabetes distress. The term diabetes distress refers to the emotional state in which individuals experience stress, guilt, or denial caused by the daily demands of living with diabetes.^16^ It is different from clinical depression, but can have similar effects on a person’s mental well-being. People with severe diabetes distress had associated matching symptoms with mild depression based on Beck Depression Inventory scores.^17^ Patients with diabetes distress avoid self-care tasks like taking medications, self-monitoring of blood glucose or attending medical appointments.^18^ Diabetes distress can also manifest as unhealthy behaviour, elevated haemoglobin A1c and impaired relationships with healthcare professionals, partners, family or friends.^5,19^ Diabetes distress prevalence rates have been reported to range from 18 to 35% among people with diabetes globally.^20^ Despite such a high burden, information on the prevalence and impact of diabetes distress among this subset of patients is minimal in many countries.

## Diabetes distress and its prevalence in different populations in India

The pooled prevalence of diabetes distress in India was estimated at 33%.^21^ However, studies conducted in India have reported a wide prevalence of diabetes distress of between 8.45 and 61.48%.^21^ The prevalence of diabetes distress among patients with type 2 diabetes in one of the states of India (Haryana, population 25 million) was 38%.^22^ A study from Chennai (population 7 million), the state capital of Tamil Nadu, India, reported the prevalence of diabetes distress to be 61.3%.^23^ However, there is relatively no data on the prevalence of diabetes distress among patients with type 2 diabetes from tier 2 cities in India (tier 2 cities are characterised by improving infrastructure, urbanisation, industrialisation and a growing middle class).

According to the Metabolic Non-communicable Disease Health Report of India – 2023, the state of Tamil Nadu in India (population: 72 million) had a 14.4% prevalence of diabetes, which is higher than the national average (11.4%).^2^ The recognition and understanding of diabetes distress by physicians in diabetes care is a crucial step toward endorsing support that is not simply prescriptive, but also person-centred and collaborative.^5^

The current study explores the burden of diabetes distress and the factors contributing to this condition among individuals living with diabetes attending a tertiary care hospital, in a tier 2 city in the state of Tamil Nadu.

## Method

The study was conducted by following the Strengthening and Reporting of Observational Studies in Epidemiology (STROBE) guidance for cross-sectional studies (Supplementary File 1 available at https://doi.org/10.1192/bjo.2026.11020).

### Survey tool

Diabetes distress was defined as a negative emotional state where people experience feelings such as stress, guilt or denial that arise from living with diabetes and the burden of self-management.^5,16^ We selected the Diabetes Distress Scale 17 (DDS-17) to assess the distress levels in the participants.^24^ The DDS-17 scale consists of 17 questions and is measured by a six-point Likert-style scoring. The DDS-17 scale measures four dimensions of distress, which are emotional burden, physician-related distress, regimen-related distress and diabetes-related interpersonal distress. The scoring ranges from 1 (never) to 6 (always) for each item (Supplementary File 2). A mean item score <2 is considered as no/low diabetes distress and a mean item score of ≥2 is considered as moderate-to-high diabetes distress. The scale was translated to Tamil (native language of Tamil Nadu) and validated (Supplementary File 3). The cut-off value for diabetes distress in the Tamil version is the same as of the original scale.

A structured questionnaire was prepared on the demographic and clinical profile, including age, gender, duration of diabetes, type of diabetes, education level, occupation, marital status, insurance coverage, type of medication intake, comorbidities (systemic hypertension, dyslipidaemia, hypothyroidism, coronary artery disease, stroke and chronic kidney disease) exercise, practising yoga, smoking and alcohol intake (as self-reported by the participants). Participants completed the questionnaire and DDS-17 scale.

### Ethical considerations

The authors assert that all procedures contributing to this work comply with the ethical standards of the Kauvery Institutional Ethics Committee (registration number ECR/966/INST/TN2017/RR-21), which is in keeping with the Helsinki Declaration of 1975, as revised in 2013. Informed written consent was secured from all participants before enrolment in the study.

### Study setting and population

Tamil Nadu is the tenth largest state in India with an area of 130 060 km^2^ and a population of 72.1 million.^25,26^ Tiruchirappalli is a major tier 2 city in Tamil Nadu, with a estimate population of 1 239 000 in 2025.^25^ The Kauvery hospital’s diabetic out-patient department caters to over 1 04 730 patients, and the diabetic diabetic out-patient department serves about 16 800 patients annually.

This prospective, observational study was conducted across a period of 3 months at the diabetic out-patient department of Kauvery Hospital, Tiruchirappalli, Tamil Nadu, India.

### Inclusion and exclusion criteria

Inclusion criteria encompassed adults aged 18 years and above, diagnosed with type 1 or type 2 diabetes or gestational diabetes, and willingness to provide informed consent. Exclusion criteria included individuals with a diagnosis or a history of a major mental health disorder such as psychosis, bipolar disorder or major clinical depression or cognitive impairment (obtained by reviewing medical history) or taking psychiatric drugs, and those not willing to participate in the study.

### Statistical analysis

Statistical analysis was performed using GraphPad PRISM software (version 11 for Windows; GraphPad, Boston, Massachusetts, USA; https://www.graphpad.com/features). Categorical variables were expressed as numbers (percentages) and continuous variables were expressed as mean ± s.d. Before applying parametric tests, data for continuous variables were tested for normality using the Shapiro–Wilk test. Variables following a normal distribution were analysed using parametric tests (Student’s *t*-test), whereas non-normally distributed variables, if any, were compared using Mann–Whitney *U*-test. Based on DDS-17 score, participants were classified into two groups: no diabetes distress group (DDS score <2) and diabetes distress group (DDS score ≥2). Differences in demographic and clinical parameters were observed between the groups. A correlation analysis was conducted to examine the relationship between the DDS-17 score and the demographic and clinical variables. Comparisons between the groups were conducted using chi-squared tests for categorical variables and *t*-tests for continuous variables. A *P*-value of <0.05 was considered statistically significant.

## Results

A total of 1250 consecutive patients were screened for the study, out of which 231 patients were excluded as they did not satisfy the inclusion criteria. The remaining 1019 patients were included in the study. Table 1 summarises the demographic characteristics and profile along with other comorbid health conditions.

**Table 1:** Patient characteristics

Characteristic	Mean ± s.d. or *n* (%)
Total population (*N*)	1019
Age (in years)	55.3 ± 12.5
Age distribution, years	
<30	25 (2.5)
30–44	163 (16)
45–59	427 (41.9)
≥60	404 (39.6)
Gender distribution	
Male	607 (59.6)
Female	412 (40.4)
Education status	
No	5 (0.5)
Higher secondary school	536 (52.6)
College	478 (46.9)
Health insurance	378 (37.1)
Married	964 (94.6)
Employment status	
Dependent	305 (30)
Retired	309 (30.4)
Working	404 (39.7)
Smoking	37 (3.6)
Alcohol	44 (4.3)
Type of diabetes	
Gestational	4 (0.4)
Type 1	11 (1.1)
Type 2	1004 (98.5)
Treatment	
Diet and tablets	770 (75.6)
Diet only	22 (2.1)
Diet, tablets and insulin	227 (22.3)
Number of comorbidities distribution	
None	386 (37.9)
One	406 (39.8)
Two	164 (16.1)
More than two	63 (6.2)
Comorbidities	
Coronary artery disease	270 (30)
Cerebrovascular disease	6 (0.6)
Dyslipidaemia	222 (24)
Systemic hypertension	339 (36.7)
Hypothyroidism	88 (9.5)
Duration of diabetes	9.6 ± 7.5
Duration of diabetes	
New onset	8 (0.7)
<1 year	66 (5.7)
1–10 years	607 (52)
11–20 years	422 (36.1)
>20 years	65 (5.6)
Number of tablets	4.2 ± 2.7
Number of tablets	
None	36 (3.5)
1–5	738 (72.4)
6–10	219 (21.5)
>10	26 (2.6)
Exercise, if yes,	
Irregular	23 (2.3)
Daily	455 (44.7)
Three times a week	202 (19.8)
No	339 (33.3)
Yoga practice	
Yes	113 (11.1)
No	906 (88.9)
DDS-17 score	1.6 ± 0.72
DDS-17 score distribution	
Less (<2)	770 (75.6)
Moderate (2–3)	178 (17.4)
Severe (>3)	71 (7.0)

DDS-17, Diabetes Distress Scale 17.

Of the included 1019 patients with diabetes, a predominant majority (98.5%, *n* = 1004) had type 2 diabetes. The average age of participants was 56 years (59.6% male, 40.4% female). Over four-fifths of respondents (81.5% *n* = 831) were over 45 years of age.

Three-quarters of the population were on lifestyle management and oral antidiabetic drugs (75.5%, *n* = 770), and just over a fifth of people were on insulin treatment (22.2%, *n* = 227). The remaining patients (*n* = 20) were on lifestyle management and not on any medication.

Nearly two-thirds of participants (62.2%, *n* = 633) had at least one or more comorbidities, with hypertension (30%, *n* = 270) and coronary artery disease (36.7%, *n* = 339) being the two most common. Just over half of the population were exercising or practising yoga daily or regularly (55.7%, *n* = 568).

Nearly a quarter of all respondents reported diabetes distress (24.4%, *n* = 249). Table 2 provides details of comparison between those with and without diabetes distress. Individuals with diabetes distress were significantly more likely to be younger (<45 years of age) compared with the no diabetes distress group (28.1 *v*. 15.3%; *P* < 0.0001). The prevalence of smoking and alcohol consumption were significantly higher among those with diabetes distress (*P <* 0.05). Individuals with diabetes distress were significantly more likely to have long-standing diabetes (>10 years) compared with the no diabetes distress group (88 *v*. 33%; *P <* 0.0001). Individuals in the diabetes distress group were significantly less likely to exercise compared with the non-diabetes distress cohort (47 *v*. 28.8%; *P* < 0.001). In this study, the term exercise refers to any planned, structured and repetitive physical activity of at least 30 minutes a day. Participants who exercised were sorted into three groups based on their frequency: daily, three times a week and irregular/inconsistent (Table 1).

**Table 2:** Comparison of characteristics between individuals with and without diabetes distress

Characteristic	No diabetes distress, mean ± s.d. or *n* (%)	Diabetes distress, mean ± s.d. or *n* (%)	*P*-value (significance at <0.05)
Total population	770 (75.6)	249 (24.4)	
Age (in years)	56.8 ± 12.3	50.8 ± 11.9	<0.0001
Age distribution, years			
<30	15 (1.9)	10 (4)	<0.0001
30–44	103 (13.4)	60 (24.1)	
45–59	307 (39.9)	120 (48.2)	
≥60	345 (44.8)	59 (23.7)	
Gender distribution			
Male	451 (58.6)	156 (62.7)	0.2542
Female	319 (41.4)	93 (37.3)	
Education status			
No	4 (0.5)	1 (0.4)	0.0425
Higher secondary school	422 (54.8)	114 (45.8)	
Graduate	344 (44.7)	134 (53.8)	
Health insurance	299 (38.8)	79 (31.7)	<0.05
Marital status			
Married	732 (95.1)	232 (93.2)	0.2464
Employment status			
Dependent	227 (29.5)	78 (31.5)	<0.0001
Retired	261 (33.9)	48 (19.4)	
Working	282 (36.6)	122 (49.2)	
Smoking	22 (2.9)	15 (6.0)	0.0232
Alcohol	27 (3.5)	17 (6.8)	0.0257
Type of diabetes			
Gestational	4 (0.5)	0 (0)	0.1398
Type 1	6 (0.8)	5 (2)	
Type 2	760 (98.7)	244 (98)	
Treatment			
Diet and tablets	598 (77.8)	172 (69.1)	0.0096
Diet only	13 (1.7)	8 (3.2)	
Diet, insulin and tablets	158 (20.5)	69 (27.7)	
Number of comorbidities distribution			
None	277 (36.1)	109 (44)	0.1157
One	316 (41.1)	88 (35.5)	
Two	129 (16.8)	34 (13.7)	
More than two	46 (6)	17 (6.9)	
Comorbidities			
Coronary artery disease	218 (30.7)	51 (25.1)	
Chronic liver disease	1 (0.1)	0 (0)	
Cerebrovascular accidents	1 (0.1)	1 (0.5)	
Dyslipidaemia	164 (23.1)	58 (28.6)	
Hypertension	242 (34)	52 (25.6)	
Systemic hypertension	26 (3.7)	20 (9.9)	
Stroke	2 (0.3)	2 (1)	
Hypothyroidism	57 (8)	19 (9.4)	
Duration of diabetes	9.7 ± 7.5	9.2 ± 7.3	0.3576
Duration of diabetes			
New onset	7 (0.9)	2 (0.8)	<0.0001
<1 year	53 (6.9)	2 (0.8)	
1–10 years	456 (59.2)	13 (5.2)	
11–20 years	203 (26.4)	151 (60.6)	
>20 years	51 (6.6)	69 (27.7)	
Number of tablets	4.2 ± 2.7	4.2 ± 2.9	>0.9999
Number of tablets			
No/1/2	20 (2.6)	16 (6.4)	<0.0001
1–5	564 (73.2)	16 (6.4)	
6–10	165 (21.4)	174 (69.9)	
>10	21 (2.7)	54 (21.7)	
Exercise			
No	222 (28.8)	117 (47)	<0.0001
Irregular	12 (1.6)	11 (4.4)	
Daily	388 (50.4)	67 (26.9)	
Three times a week/yes	148 (19.2)	54 (21.7)	
Yoga practice	93 (12.1)	20 (8.0)	0.5961

A negative correlation was identified between diabetes distress score on the DDS-17, age (*r* = −0.2365; *P* < 0.0001) and number of comorbidities (*r* = −0.07363; *P =* 0.0196) (Table 3).

**Table 3:** Correlation analysis between DDS-17 score and demographic/clinical variables

Comparison	*r*-value	*P*-value (significance at <0.05)
DDS-17 versus age	−0.2365	<0.0001
DDS-17 versus diabetes duration	−0.01099	0.7273
DDS-17 versus gender	0.02038	0.5159
DDS-17 versus number of tablets	−0.01273	0.6890
DDS-17 versus number of comorbidities	−0.07363	0.0196
DDS-17 versus emotional burden	0.8627	<0.0001
DDS-17 versus physician-related distress	0.1021	0.0011
DDS-17 versus regimen-related distress	0.7760	<0.0001
DDS-17 versus diabetes-related interpersonal distress	0.7064	<0.0001
DDS-17 versus yoga practice	−0.07177	0.0219

DDS-17, Diabetes Distress Scale 17.

**Fig. 1:**
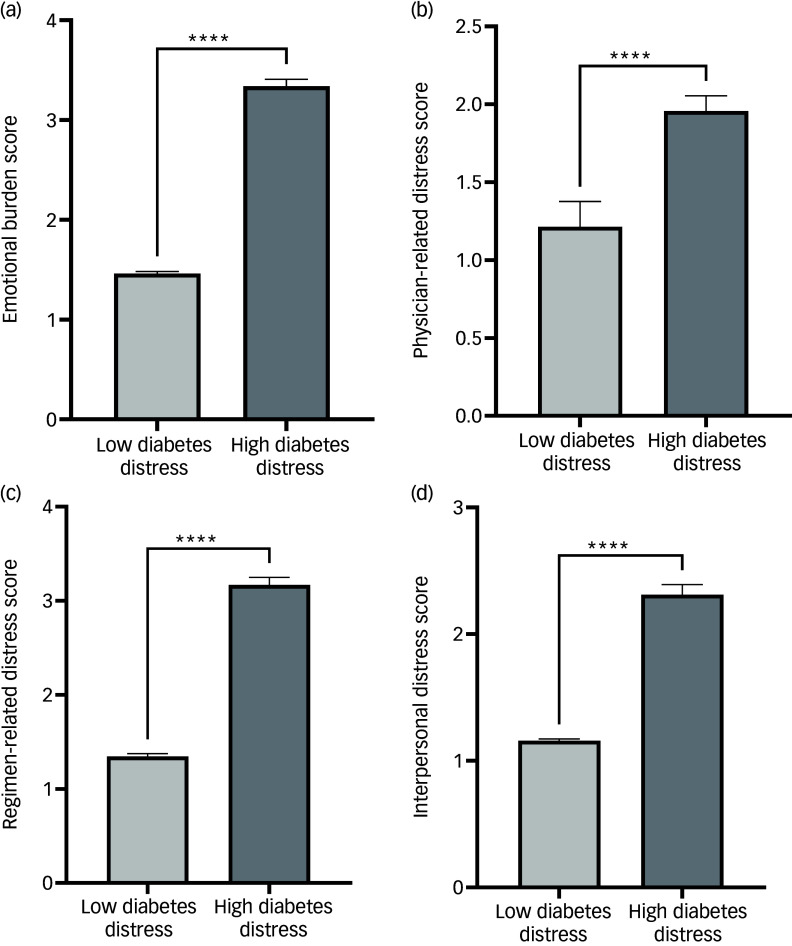
Comparison of emotional burden, physician-related distress, regimen-related distress and diabetes-related interpersonal distress scores between patients with type 2 diabetes with and without diabetes distress, according to DDS-17 scores. DDS-17, Diabetes Distress Scale 17. ****P <0.0001.

The subdomain scores of emotional burden, physician-related distress, regimen-related distress and diabetes-related interpersonal distress were all significantly higher among those with diabetes distress than those with no diabetes distress (Fig. 1). A significant positive correlation was observed between diabetes distress scores and domains of emotional burden (*r* = 0.8627; *P* < 0.0001*)*, physician-related distress (*r* = 0.1021; *P* = 0.0011), regimen-related distress (*r* = 0.7760; *P* < 0.0001) and diabetes-related interpersonal distress (*r* = 0.7064; *P* < 0.0001) (Table 3).

## Discussion

Our study highlights the high prevalence of psychological distress in the form of diabetes distress, and its associated factors, in middle-aged patients with type 2 diabetes in a tier 2 city in India. Our study observed the prevalence of diabetes distress in about a fourth of the study population compared with the 33% pooled prevalence of diabetes distress identified by a systematic review and meta-analysis pooled prevalence of diabetes distress of Indian patients with diabetes.^21^ It could be that the discrepancies in economic and health care resources across India play a role in the heterogeneity of prevalence findings. However, it still highlights the significant burden diabetes distress brings to people with diabetes in India. Addressing diabetes distress is critical for improving self-management and health outcomes among individuals with diabetes.

One finding of our study was that individuals with diabetes distress had long-standing diabetes. A study (*N* = 142) done in another region of India, using the same DDS-17 and study duration, showed a significant increase in the total diabetes distress scores and the subscores with increasing diabetes duration as seen in our study.^27^

Another aspect of our findings was that those with diabetes distress are relatively younger. Similarly, a study (*N* = 438) found nearly a quarter (24%) of individuals with young-onset diabetes reported high levels of diabetes distress, including emotional burden and regimen-related distress.^28^ Young adults often grapple with feelings of being overwhelmed, anxious and defeated because of the constant demands of managing their condition. Factors contributing to this distress include regimen-related concerns, emotional burdens and interpersonal issues related to diabetes management.^29,30^ All of these were supportive of our findings in relation to the subdomains of the DDS-17. In addition, young individuals may struggle with dietary restrictions that interfere with social activities or career advancement opportunities, leading to feelings of isolation and frustration. Young adults are typically navigating significant life transition such as pursuing education or starting career while simultaneously managing a chronic condition.^12^ Young adults experiencing diabetes distress often report higher haemoglobin A1c levels, indicating suboptimal blood glucose management and increased risk of comorbidities.^21,28,29^

Patients on more complicated regimens often report feeling overwhelmed, which can lead to poor adherence to treatment protocols and ultimately worsen their glycaemic control.^30^ The analysis in our study revealed that individuals with diabetes distress had a higher number of medications compared with those with no or low diabetes distress. Studies have shown that emotional distress, including diabetes distress, is a significant predictor of medication adherence behaviour among patients with type 2 diabetes.^31^ Those experiencing high levels of distress are less likely to adhere to their prescribed medication regimens, which can result in a cycle of worsening health outcomes and increased medication needs.^31,32^

With urbanisation and industrial development, there has been an increase in sedentary lifestyle in India, especially given the growth of number of tier 2 cites.^33^ A third of individuals with diabetes distress in our study reported that they did not exercise. Sedentary lifestyle is attributed to various health complications, including cardiovascular disease and risk for mortality. Individuals who sit for long periods without adequate physical activity face a 73% higher risk of early death compared with their more active counterparts.^34^ Therefore, engaging in regular physical activity not only helps mitigate these risks, but also contributes to improved mental health and overall well-being, reinforcing the importance of movement in daily routines.

Our study suggests that as diabetes distress increases, the emotional burden experienced by individuals also intensifies. This relationship underscores the psychological impact of diabetes management, where the demands of self-care can lead to heightened feelings of stress and anxiety. These correlations further illustrate how diabetes distress is intricately linked to the practical aspects of daily diabetes management, including treatment regimens and the overall impact on one’s life.

### Limitations

Our study is a cross-sectional study, and thus has the limitations that such cross-sectional studies bring in principle, in that they focus on association and not causation. We cannot claim our sample to be representative of the diabetes population, as the participants belonged to a single centre. Therefore, the results may not be able to be applied to broader populations because of differences in demographic and social factors. This could be considered a convenience sample. Additionally, the absence of biochemical parameters to determine the extent of glycaemic and blood pressure control and lipid levels limits the understanding of the relationship between physiological health and diabetes distress. Additionally, by excluding individuals with a history of diagnosed mental health disorders and/or cognitive impairments, we cannot determine if some people in the excluded cohort had a higher psychological burden of diabetes distress and who may be at higher risk of diabetes distress by virtue of their pre-existing mental health and/or cognitive problems.

### Future implications

Our study suggests that younger individuals are more likely to experience diabetes distress. This subset of people should be regularly screened and appropriately treated for reducing the psychological burden and to promote generalised well-being. We suggest large-scale interventional studies to further clarify the risk factors associated with diabetes distress.

In summary, our study is a relatively large-scale study of >1000 participants selected continuously (subject to inclusion criteria being met), highlighting, for the first time, the association of psychological distress with diabetes in a tier 2 city hosting an emergent middle-class urban population. The high burden of diabetes distress highlighted in our study underscores the critical need for a holistic approach to diabetes management. There is a need to recognise the emotional and psychological challenges faced by individuals with diabetes by healthcare providers, and develop comprehensive care strategies that address both physical and mental health. This integrated approach may improve clinical outcomes and foster a supportive environment that prioritises the overall well-being of individuals with diabetes. Ultimately, these strategies can lead to enhanced quality of life and better adherence to diabetes management plans, paving the way for more effective and compassionate care.

## Supporting information

10.1192/bjo.2026.11020.sm001 Palaniswamy et al. supplementary material 1 Palaniswamy et al. supplementary material

10.1192/bjo.2026.11020.sm002 Palaniswamy et al. supplementary material 2 Palaniswamy et al. supplementary material

10.1192/bjo.2026.11020.sm003 Palaniswamy et al. supplementary material 3 Palaniswamy et al. supplementary material

## Data Availability

The data that support the findings of this study are available from the corresponding author, R.S., upon reasonable request.

## Supplementary material

The supplementary material is available online at https://doi.org/10.1192/bjo.2026.11020
