# Prevalence and determinants of diabetes-related psychological distress in a tertiary care setting in Tamil Nadu, India: cross-sectional study – CORRIGENDUM

**DOI:** 10.1192/bjo.2026.12037

**Published:** 2026-06-02

**Authors:** Gowri Palaniswamy, Lawrence Soosai Nathan, Senthil R. Kumar, Aravindakumar Subramanium, Karthikeyan Aravindakumar Gowri, Dhasaratharaman Thirunavukkarasu, Samuel J. Tromans, Rohit Shankar

In the original publication of this Paper, author Gowri Palaniswamy’s name was incorrectly given as ‘Gowri Aravind’.

This has since been updated in the article.

The authors apologise for this error.
