# Supplementary material for: Prevalence and determinants of diabetes-related psychological distress in a tertiary care setting in Tamil Nadu, India: cross-sectional study
Source: BJPsych Open. 2026 Apr 27;12(3):e119. doi: 10.1192/bjo.2026.11020 (PMC13122335; doi:10.1192/bjo.2026.11020)
Supplement: Palaniswamy et al. supplementary material 2 — Palaniswamy et al. supplementary material [file S2056472426110205sup002.docx]

**Supplementary information 2: Diabetes Distress Scale –17**

The measurement scale for responses to DDS-17 questionnaire

| Not a Problem – 1 |
| --- |
| A Slight Problem – 2 |
| A Moderate Problem – 3 |
| Somewhat Serious Problem – 4 |
| A Serious Problem – 5 |
| A Very Serious Problem – 6 |

| **DDS-17 questionnaire** |
| --- |
| Q1 Feeling that diabetes is taking up too much of my mental and physical energy every day |
| Q2 Feeling that my doctor doesn’t know enough about diabetes and diabetes care |
| Q3 Feeling angry, scared and/or depressed when I think about living with diabetes |
| Q4 Feeling that my doctor doesn’t give me clear enough directions on how to manage my diabetes |
| Q5 Feeling that I am not testing my blood sugars frequently enough |
| Q6 Feeling that I am often failing with my diabetes routine |
| Q7 Feeling that friends or family are not supportive enough of self-care efforts (e.g. planning activities that conflict with my schedule, encouraging me to eat the “wrong” foods) |
| Q8 Feeling that diabetes controls my life |
| Q9 Feeling that my doctor doesn’t take my concerns seriously enough |
| Q10 Not feeling confident in my day-to-day ability to manage diabetes |
| Q11 Feeling that I will end up with serious long-term complications, no matter what I do |
| Q12 Feeling that I am not sticking closely enough to a good meal plan |
| Q13 Feeling that friends or family don’t appreciate how difficult living with diabetes can be |
| Q14 Feeling overwhelmed by the demands of living with diabetes |
| Q15 Feeling that I don’t have a doctor, who I can see regularly enough about my diabetes |
| Q16 Not feeling motivated to keep up my diabetes self-management |
| Q17 Feeling that friends or family don’t give me the emotional support that I would like |

Interpretations of DDS sore

1. *Total DDS Score:*

To determine the degree of distress,

DDS Score – Total score of diabetes distress/ 17

A mean score ≥ 3 is diagnostic of severe diabetes distress.

1. *To estimate emotional burden:*

Sum of 5 questions (Q1,3,8,11,4) from DDS-17 questionnaire is considered:

Emotional burden DDS Score – Total score of diabetes distress/ 5

A mean score ≥ 3 is diagnostic of severe emotional burden-related diabetes distress.

1. *To estimate physician-related distress:*

Sum of 4 questions (Q 2,4,9,15) from DDS-17 questionnaire is considered:

Physician-related distress DDS Score – Total score of diabetes distress/ 4

A mean score ≥ 3 is diagnostic of severe Physician-related diabetes distress.

1. *To estimate Regimen-related Distress:*

Sum of 5 questions (Q 5,6,10,12,16) from DDS-17 questionnaire is considered:

Regimen-related Distress DDS Score – Total score of diabetes distress/ 5

A mean score ≥ 3 is diagnostic of severe Regimen-related diabetes distress.

1. *To estimate Interpersonal Distress:*

Sum of 3 questions (Q 7,13,17) from DDS-17 questionnaire is considered:

Interpersonal Distress DDS Score – Total score of diabetes distress/3

A mean score ≥ 3 is diagnostic of severe Interpersonal diabetes distress.
