# Supplementary material for: Prevalence and determinants of diabetes-related psychological distress in a tertiary care setting in Tamil Nadu, India: cross-sectional study
Source: BJPsych Open. 2026 Apr 27;12(3):e119. doi: 10.1192/bjo.2026.11020 (PMC13122335; doi:10.1192/bjo.2026.11020)
Supplement: Palaniswamy et al. supplementary material 3 — Palaniswamy et al. supplementary material [file S2056472426110205sup003.docx]

**Supplementary 3: Translation and Validation process for the Diabetes Distress Scale: Tamil Version**

The Diabetes Distress scale was translated from English to Tamil using forward-backward translation procedures following WHO guidelines. An expert panel of four members including a diabetologist, general practitioner, language specialist and a psychologist reviewed the translations to ensure conceptual equivalence, cultural appropriateness and clarity. After expert committee review, a pilot study was done with 10 Tamil speaking individuals with diabetes to assess clarity, understanding and cultural relevance. Psychometric validation of the DDS (Tamil) was done with 250 adult Type 2 diabetic participants, which showed good internal consistency (Cronbach’s alpha=0.91) and test-retest reliability (Intraclass Correlation Coefficient =0.91) significant at p<0.000). DDS (Tamil) had a significant correlation with HbA1c (p=0.70, p<0.000). Exploratory factor analysis supported the original scale structure, confirming the Tamil version as a valid and reliable tool for assessing diabetes-related distress in Tamil-speaking populations.
